# Adjuvant treatment recommendations for patients with ER-positive/HER2-negative early breast cancer by Swiss tumor boards using the 21-gene recurrence score (SAKK 26/10)

**DOI:** 10.1186/s12885-017-3261-1

**Published:** 2017-04-13

**Authors:** Bernhard C. Pestalozzi, Christoph Tausch, Konstantin J. Dedes, Christoph Rochlitz, Stefan Zimmermann, Roger von Moos, Ralph Winterhalder, Thomas Ruhstaller, Andreas Mueller, Katharina Buser, Markus Borner, Urban Novak, Catrina Uhlmann Nussbaum, Bettina Seifert, Martin Bigler, Vincent Bize, Simona Berardi Vilei, Christoph Rageth, Stefan Aebi, The Swiss Group for Clinical Cancer Research (SAKK)

**Affiliations:** 1 Universitaetsspital Zuerich, Raemistrasse 100, 8091 Zurich, Switzerland; 2 Brustzentrum Zuerich, Zurich, Switzerland; 3 Universitaetsspital Basel, Basel, Switzerland; 4 Hôpital Cantonal Fribourg, Fribourg, Switzerland; 5 Kantonsspital Graubuenden Chur, Chur, Switzerland; 6 Luzerner Kantonsspital, Lucerne, Switzerland; 7 Breast Center St. Gallen, St. Gallen, Switzerland; 8 Kantonsspital Winterthur, Winterthur, Switzerland; 9 Engeriedspital Bern, Bern, Switzerland; 10 Spitalzentrum Biel, Biel, Switzerland; 11 Inselspital Bern, Bern, Switzerland; 12 Kantonsspital Olten, Olten, Switzerland; 13 Kantonsspital Baselland, Liestal, Switzerland; 14 SAKK Coordinating Center, Bern, Switzerland

**Keywords:** ER-positive early breast cancer, Adjuvant treatment recommendation, Multigene expression profiling, Recurrence score, Oncotype DX

## Abstract

**Background:**

To evaluate the effect of Recurrence Score® results (RS; Onco*type* DX® multigene assay ODX) on treatment recommendations by Swiss multidisciplinary tumor boards (TB).

**Methods:**

SAKK 26/10 is a multicenter, prospective cohort study of early breast cancer patients: Eligibility: R0-resection, ≥10% ER+ malignant cells, HER2–, pN0/pN1a. Patients were stratified into low-risk (LR) and non-low-risk (NLR) groups based on involved nodes (0 vs 1–3) and five additional predefined risk factors. Recommendations were classified as hormonal therapy (HT) or chemotherapy plus HT (CT + HT). Investigators were blinded to the statistical analysis plan. A 5%/10% rate of recommendation change in LR/NLR groups, respectively, was assumed independently of RS (null hypotheses).

**Results:**

Two hundred twenty two evaluable patients from 18 centers had TB recommendations before and after consideration of the RS result. A recommendation change occurred in 45 patients (23/154 (15%, 95% CI 10–22%) in the LR group and 22/68 (32%, 95% CI 22–45%) in the NLR group). In both groups the null hypothesis could be rejected (both *p* < 0.001). Specifically, in the LR group, only 5/113 (4%, 95% CI 1–10%) with HT had a recommendation change to CT + HT after consideration of the RS, while 18/41 (44%, 95% CI 28–60%) of patients initially recommended CT + HT were subsequently recommended only HT. In the NLR group, 3/19 (16%, 95% CI 3–40%) patients were changed from HT to CT + HT, while 19/48 (40%, 95% CI 26–55%) were changed from CT + HT to HT.

**Conclusion:**

There was a significant impact of using the RS in the LR and the NLR group but only 4% of LR patients initially considered for HT had a recommendation change (RC); therefore these patients could forgo ODX testing. A RC was more likely for NLR patients considered for HT. Patients considered for HT + CT have the highest likelihood of a RC based on RS.

## Background

While initial trials in unselected women with early-stage ER-positive breast cancer failed to demonstrate a beneficial effect from adding chemotherapy to adjuvant endocrine therapy [1–4], it later became apparent that adjuvant chemo-endocrine therapy does reduce the recurrence rate compared with adjuvant endocrine therapy alone in certain selected populations. One approach to identifying which patients should receive adjuvant CT in addition to adjuvant endocrine therapy was described in the St. Gallen Consensus Highlights in 2009 [5]. The following factors can be used to separate patients with lower and higher risk of relapse and death: Nodal status, primary tumor size, ER/PgR level, histologic tumor grade, proliferation fraction (Ki67), lymphovascular invasion. In addition, the NSABP B-20 [6] and SWOG 8814 [7] trials demonstrated that gene expression profiling is a useful tool for selecting patients who are most likely to benefit from adding chemotherapy to adjuvant treatment in patients with node-negative and node-positive breast cancers, respectively. In both these trials, patients were stratified into three distinct groups following measurement of RNA expression using the ODX assay developed by Genomic Health Incorporated (GHI). The ODX assay is a multigene reverse-transcriptase polymerase chain reaction (RT-PCR) test that analyzes the expression of 21 genes and estimates the 10-year distant breast cancer recurrence risk. The assay was validated in the NSABP B-14 trial, a large, multicenter trial for women with node-negative, ER-positive breast cancer treated with tamoxifen [8]. The ODX assay result Recurrence Score (RS) provides as a continuous variable ranging from 0 to 100. For statistical analysis, patients are typically grouped into three risk categories based on the RS: low (RS 0–17), intermediate (RS 18–30), and high (RS ≥31). These categories have been shown to correlate with the rate of distant recurrence in multiple studies in node negative [8–11] and node positive disease [7, 12] at 10 years as well as overall survival [8].

The NSABP B-20 trial showed that the benefit of adding chemotherapy (cyclophosphamide, methotrexate, fluorouracil) to tamoxifen was mainly seen in a relatively small group of patients with a high RS (≥31) [6]. These patients had an absolute decrease in 10-year distant recurrence rate of 28% with adjuvant chemotherapy plus endocrine therapy compared with adjuvant endocrine therapy alone. Patients in the low and intermediate RS risk groups had no significant benefit from the addition of chemotherapy [6]. Similarly, in the SWOG 8814 (INT0100) trial performed in patients with node-positive breast cancer the benefit of adding cyclophosphamide, doxorubicin, and fluorouracil followed by tamoxifen compared with tamoxifen alone was seen only in patients with high RS [7].

The ODX assay has been available in the US since 2004 as a tool to aid a physician’s treatment recommendation for patients with early breast cancer and was recommended by an ASCO expert panel for use in patients with ER-positive early breast cancer in 2007 [13]. TAILORx, a prospective clinical trial, confirmed that patients with node negative breast cancer and a low RS (<11) had a very low risk of distant relapse [11].

Several studies have attempted to analyze the contribution of ODX assay to decisions on adjuvant treatment in patients with ER-positive breast cancer. In an American study of 89 patients with node-negative disease, the assay led to changes in treatment decisions in 31% of cases [14], although whether design of this study allowed these decision changes to be attributed to the use of the ODX assay was later questioned [15]. Since then, similar studies in Australia [16], Canada [17], Germany [18], France [19], Israel [20], Japan [21], Spain [22], and the United Kingdom [23] have consistently shown that knowledge of the ODX RS changed treatment plans in about 30% of cases. The majority of changed decisions were due to “de-escalation” from chemotherapy plus endocrine therapy to endocrine therapy alone.

In Switzerland, the ODX assay was not reimbursed until 2015 and was rarely performed. In contrast, Swiss oncologists often used the Ki-67 proliferation assay, expression levels of estrogen and/or progesterone receptor, histological grading and nodal status to support a recommendation for or against adjuvant chemotherapy [24, 25].We studied whether the introduction of the ODX assay impacted on treatment recommendations issued by Swiss multidisciplinary tumor boards.

## Methods

### Patient population

SAKK 26/10 (NCT01926964) is a multicenter, prospective cohort study. Participants had completely resected breast cancer with pathologically confirmed negative margins. Participants also had to have ≥10% ER-positive invasive malignant cells, HER2-negative carcinoma by immunohistochemistry (0 or 1+) or by FISH (ratio of HER2/Cen17 ≤ 2.0) by local pathology, and a regional lymph node status of pN0 or pN1a (1–3 positive nodes) as determined by axillary dissection or sentinel procedure [26, 27].

Each case was presented at the center’s multidisciplinary tumor board where a first recommendation on adjuvant systemic treatment was issued. The following items were required for baseline data collection (“pre-registration” step): pathologic maximum tumor diameter (in mm), percentage of ER-positive and progesterone (PgR)-positive invasive tumor cells, Ki-67 proliferation rate (MIB-1 antibody), and the modified Bloom-Richardson-Elston (BRE) grade. In addition, for study eligibility (“registration” step), the patient had to be considered suitable to receive adjuvant chemotherapy, have a WHO performance status 0–1, and have sufficient invasive breast cancer tissue available to prepare 39 tissue sections (thickness 5 μm). Patients with bilateral invasive breast cancer and those with tumors of stage cT4, pT4 or pN ≥ 2 or known metastatic breast cancer were excluded, as were pregnant women and patients with psychiatric or medical diagnoses potentially interfering with their ability to give informed consent.

### Study procedures

The treating physician then invited the patient to participate in the study, explaining in the discussion the uncertainty surrounding the use of adjuvant chemotherapy in addition to the adjuvant endocrine therapy, and issuing the recommendation of the first tumor board discussion. If the patient agreed to participate in the study, the result of this first discussion was recorded as the “first shared decision”. Only after this, breast cancer tissue was sent to Genomic Health Inc. where the ODX RS was determined and returned usually within 7 working days after receipt of the samples. The patient case was then re-presented at the tumor board along with the RS, and a second tumor board recommendation was issued. The RS result and the second tumor board recommendation were then discussed with the patient, and the result of this “second shared decision” was recorded. The “treatment actually given” after the initiation of adjuvant systemic treatment was also recorded. All recommendations (i.e. first tumor board recommendation, first shared decision, second tumor board recommendation, second shared decision) as well as the treatment actually given were classified as either hormonal therapy (HT) or chemotherapy plus endocrine therapy (CT + HT).

The primary endpoint of the study was the percentage of treatment decisions that changed between the first and second tumor board recommendations (after the inclusion of the RS). Importantly, the investigators contributing patients to the study were blinded to the statistical analysis plan.

The protocol also stratified the patients into two risk groups based on involved nodes (0 versus 1–3) and other predefined risk factors taken from the St. Gallen consensus guidelines [5, 29]. These risk factors were: ER-positivity <50% and PgR-positivity <50%, grade 3, tumor size >5 cm, extensive lympho-vascular invasion, and Ki67 > 30%. Patients were deemed to be at low risk of recurrence if they had pN0 tumor and ≤1 risk factor present or pN1a with no other risk factor. Patients were non-low risk if they had pN0 tumor plus ≥2 risk factors, or if pN1a plus ≥1 other risk factor. This risk stratification was held concealed from the participating investigators.

### Statistical analyses

The null hypothesis was that there would be a 5% change of recommendation in the low-risk group, and a 10% change of recommendation in the non-low-risk groups, following the second tumor board recommendation. These levels of change were expected to occur independently of the RS. The alternative hypothesis was that a change of recommendation after consideration of the RS would occur in at least 15% in the low-risk group, and of at least 25% in the non-low-risk group. These levels of change were set a priori to be considered clinically interesting and relevant. Using these hypotheses and the exact binomial test, assuming a one-sided type I error of 0.025, a power of 90%, and an expected proportion of non-evaluable patients of 10%, the required sample size was 175 patients in total (93 patients in the low-risk group, 64 patients in the non-low-risk group). When the low-risk group had attained its accrual goal it was decided to continue accrual into both groups in order to keep up the blinding of investigators concerning the concealed statistical analysis plan. The study was closed only when the non-low-risk group was complete.

Point estimates and the corresponding exact 95% confidence interval were calculated for proportions. Spearman’s rank correlation coefficient together with the 95% confidence interval was calculated for the comparison of scores, while the kappa coefficient with the 95% confidence interval was calculated to compare categorized scores. SAS 9.2 and R 3.1.0 were used for the statistical analyses.

## Results

**Fig. 1:**
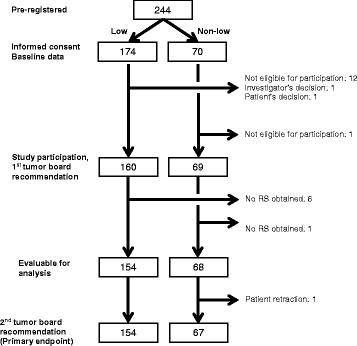
Patient Flow

Between July 2013 and June 2014 we recruited 229 eligible patients at 18 study sites in Switzerland. Of these, 222 patients were evaluable and 221 patients had a recommendation from both the first and second tumor board and had an ODX RS available (Fig. 1): 154 patients in the low-risk group and 67 patients in the non-low-risk group.

Patient characteristics of all 229 patients entered into the study are shown in Table 1. Median patient age was 58 years (range 32 to 82). Two thirds of the patients were postmenopausal, pT-stage was mostly T1b, T1c or T2, 21% of the patients had grade 3 tumors and 22% had peritumoral lympho-vascular invasion. A total of 88 (38%) of all patients had 1–3 positive nodes. Histologic type was mostly invasive ductal carcinoma. Risk factors and their distribution among risk groups for the evaluable patients are shown in Table 2. The distribution of ODX low-, intermediate-, and high-risk RS was 65% (100 of 154), 31% (48 of 154), and 4% (6 of 154) in the low-risk group and 51% (34 of 67), 28% (19 of 67), and 21% (14 of 67) in the non-low-risk group, respectively.

**Table 1:** Patient characteristics

Variable	Low-risk group (*N* = 160)	Non-low-risk group (*N* = 69)	Total (*N* = 229)
Age, median (range)	58 (35–82)	58 (32–79)	58 (32–82)
Menopausal status, *n* (%)			
Premenopausal	41 (26%)	23 (33%)	64 (28%)
Peri-menopausal	5 (3%)	5 (7%)	10 (4%)
Postmenopausal	114 (71%)	41 (59%)	155 (68%)
pT stage, *n* (%)			
T1	2 (1%)	2 (3%)	4 (2%)
T1a	2 (1%)	-	2 (1%)
T1b	19 (12%)	5 (7%)	24 (10%)
T1c	75 (47%)	33 (48%)	108 (47%)
T2	57 (36%)	23 (33%)	80 (35%)
T3	4 (3%)	6 (9%)	10 (4%)
Tis	1 (1%)	-	1 (0%)
pN stage, *n* (%)			
pN0	122 (76%)	19 (28%)	141 (62%)
pN1a	38 (24%)	50 (72%)	88 (38%)
Histologic type, n (%)			
Invasive ductal carcinoma	119 (74%)	56 (81%)	175 (76%)
Invasive lobular carcinoma	31 (19%)	9 (13%)	40 (17%)
Other	10 (6%)	4 (6%)	14 (6%)
Tumor grade (BRE), *n* (%)			
G 1	24 (15%)	5 (7%)	29 (13%)
G 2	124 (78%)	27 (39%)	151 (66%)
G 3	12 (8%)	37 (54%)	49 (21%)
Peritumoral lympho-vascular invasion, *n* (%)			
No	153 (96%)	25 (36%)	178 (78%)
Yes	7 (4%)	44 (64%)	51 (22%)

**Table 2:** Distribution of predefined risk factors in the low- and non-low-risk groups (all evaluable patients)

Variable	Low-risk group^a^ (*N* = 154)	Non-low-risk group^a^ (*N* = 68)^b^	Total (*N* = 222)
N0	117 (76%)	19 (28%)	136 (61%)
N1a	37 (24%)	49 (72%)	86 (39%)
pT3	3 (2%)	6 (9%)	9 (4%)
Grade 3	12 (8%)	36 (53%)	48 (22%)
Lympho-vascular invasion	7 (5%)	43 (63%)	50 (23%)
ER <50% and PgR < 50%	–	1 (1%)	1 (0%)
Ki67 > 30%	10 (6%)	19 (28%)	29 (13%)
Risk factors, mean (range)	0.2 (0–1)	1.6 (1–3)	0.6 (0–3)

Data are *n* (%) unless otherwise indicated

^a^Definitions. Low-risk: N0 and ≤1 predefined risk factor, or N1a with no predefined risk factor. Non-low-risk: N0 and ≥2 predefined risk factors, or N1a with ≥1 predefined risk factors (Comment: These classifications were fixed in the protocol but not disclosed to study participants)

^b^One evaluable patient withdrew from the study before the second tumor board provided a recommendation

**Fig. 2:**
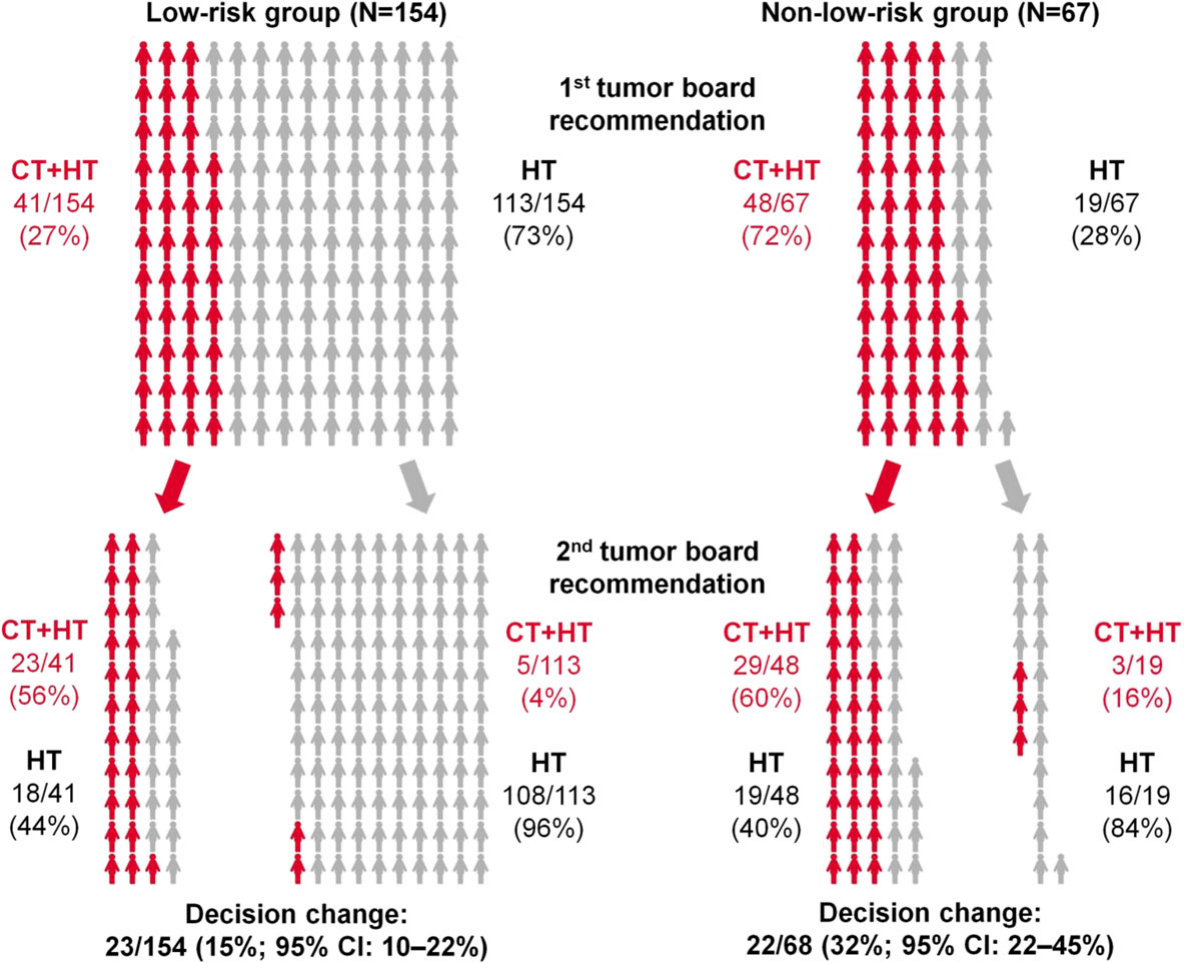
Primary endpoint: Change in adjuvant treatment recommendation between the first and second tumor board (after knowledge of the ODX recurrence score)

In the 222 patients evaluable for the primary endpoint, the recommendations of the first and second tumor board differed in 45 (20%) patients: 23 of 154 (15%, 95% CI 10–22%) in the low-risk group, and 22 of 68 (32%, 95% CI 22–45%) in the non-low-risk group (Fig. 2). Therefore, in both groups the null hypothesis (5% change in the low risk, 10% change in the non-low risk group) was rejected at the 0.025 level. The *p*-value was <0.001 in both groups. Specifically, in the low-risk group, five of 113 (4%, 95% CI 1–10%) patients with an initial HT recommendation were changed to CT + HT, while 18 of 41 (44%, 95% CI 28–60%) of patients with an initial CT + HT recommendation were changed to HT. In the non-low-risk group, three of 19 (16%, 95% CI 3–40%) patients were changed from HT to CT + HT, while 19 of 48 (40%, 95% CI 26–55%) were changed from CH + HT to HT.

Characteristics of the 8 patients with a change in recommendation from HT to CT + HT can be found in the Appendix (Table 7). Most notably for these 8 patients the median RS (range) was 30 (18–51), showing that RS was a very prominent factor for these 8 decision changes, since half the patients were in the high and the other half in the intermediate risk RS group.

We also investigated how the recommendations of the tumor boards translated into actual patient care. The “evolution” of treatment recommendations from the first tumor board recommendation to the treatment actually given is shown in Table 3. In addition, we have analyzed the reasons for decision changes in the low-risk/non-low-risk groups (Table 4): Most treatment decision changes were due to the knowledge of RS at the second tumor board (Table 4).

**Table 3:** Evolution of treatment recommendations

Risk category	Low-risk group (*N* = 154)		Non-low-risk group (*N* = 67)	
Recommendation, n	CT + HT	HT	CT + HT	HT
1st tumor board	41 (27%)	113 (73%)	48 (72%)	19 (28%)
1st shared decision	40 (26%)	114 (74%)	47 (70%)	20 (30%)
	Add knowledge of recurrence score			
2nd tumor board	28 (18%)	126 (82%)	32 (48%)	35 (52%)
2nd shared decision	24 (16%)	130 (84%)	32 (48%)	35 (52%)
Treatment actually given	23 (15%)	130 (84%)	28 (42%)	37 (55%)

*CT* chemotherapy, *HT* endocrine therapy

**Table 4:** Reason for change of recommendation

	Low-risk group (*N* = 154)	Non-low-risk group (*N* = 67)
Number of patients with decision change, n	23	22
Reasons^a^ for decision change, *n*:		
Recurrence score	23 (100%)	21 (95%)
Opinion of tumor board changed	1 (4%)	1 (5%)
Patient preference	3 (13%)	1 (5%)
Other	2 (9%)	1 (5%)

^a^More than one reason for changing the treatment decision were possible

Further factors analyzed and never found to be a reason for change of recommendation after second tumor board were: Tumor board composition change, new medical information

Recommendations of the second tumor board that included knowledge of RS are shown in Table 5. All patients with a high-risk RS (31–100) were recommended to receive CT + HT. Most (124 of 134; 93%) but not all of the patients with a low RS (0–17) were offered HT only. In the patients with intermediate RS (18–30) tumor board recommendations were variable. Treatment recommendations changed in 22% of patients with RS 0–17; 16% of patients with RS 18–30; and 20% of patients with RS 31–100.

**Table 5:** Distribution of second tumor board recommendations according to the recurrence score

Risk category	Low-risk group (*N* = 154)		Non-low-risk group (*N* = 67)		Total
	CT + HT	HT	CT + HT	HT	
All recommendations	28	126	32	35	221
RS 0–17 (low)	4	96	6	28	134 (61%)
RS 18–30 (intermediate)	18	30	12	7	67 (30%)
RS 31–100 (high)	6	0	14	0	20 (9%)

**Fig. 3:**
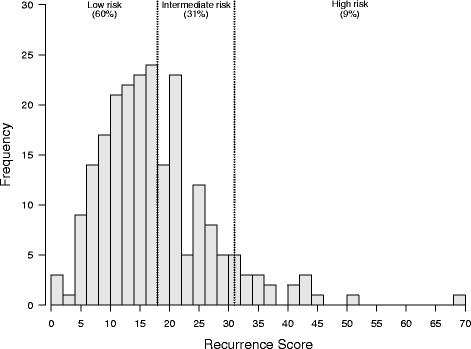
Distribution of Recurrence Score

The distribution of RS overall as well as for different subgroups can be found in Table 6. A graphic presentation of all the recurrence scores is shown in Fig. 3.

**Table 6:** Distribution of RS overall and for different subgroups

Variable	RS median (range)
All patients, *N* = 222	16 (0–68)
Tumor grade (BRE)	
G1, *N* = 28	14 (6–28)
G2, *N* = 146	15 (0–51)
G3, *N* = 48	24 (5–68)
pN stage	
pN0, *N* = 136	17 (0–68)
pN1a, *N* = 86	14 (0–51)
Peritumoral lympho-vascular invasion	
No, *N* = 172	16 (0–68)
Yes, *N* = 50	15 (2–44)
Invasive tumor size	
≤ 2 cm, *N* = 133	15 (0–43)
> 2 cm, *N* = 89	16 (0–68)
Ki67	
≤ 30, *N* = 193	15 (0–51)
> 30, *N* = 29	24 (1–68)

## Discussion

In this multicenter, prospective cohort study of early breast cancer patients we studied how Swiss tumor boards made use of the ODX 21-gene multigene expression assay RS. This assay may be considered for routine use in the USA for all patients with ER-positive/HER2-negative tumors of at least 5 mm diameter (T1b and larger) [26]. Our findings suggest that this assay may be used in a more judicious manner, as opposed to testing all patients with early stage ER-positive/HER2-negative breast cancer. We found that knowledge of the RS resulted in a very low rate of decision change for patients initially offered HT only, i.e. 5 of 113 (4%, 95% CI 1–10%) in the low-risk group, and 3 of 19 (16%, 95% CI 3–40%) in the non-low-risk group. We therefore think that it is reasonable for these patients to forgo additional testing with the ODX assay, particularly for the patients fulfilling the low-risk criteria (Table 2). Obviously, this statement has to be tempered by the fact that the confidence intervals are large, and that the primary decision to recommend HT only is subjective and may not be applicable to other centers. On the other hand, patients initially considered for CT + HT have a high likelihood of decision change based on the knowledge of the RS, with 18 of 41 (44%) in the low-risk and 19 of 48 (40%) in the non-low-risk group. These patients at non low-risk as defined by our criteria may be considered good candidates to have ODX testing.

Overall, our data show that the ODX assay, when readily available, significantly impacts adjuvant treatment decisions in patients with ER-positive/HER2-negative early breast cancer. Changes in treatment recommendations at the second tumor board were predominantly driven by the availability of the RS. When considering the entire study population treatment decisions changed in approximately 20% of patients, which is somewhat lower than the level of change seen in other studies [9, 11–18]. A recent meta-analysis of 15 studies that investigated the impact of the ODX assay on adjuvant treatment decisions reported that the additional information provided by the RS changed the recommendation for adjuvant treatment in 30% of cases [30]. The lower rate of decision changes in our study may reflect the fact that the assay was not applied to all patients who satisfied the inclusion criteria. Investigators were free not to offer study participation to patients. Investigators were also free in their interpretation of the RS which leaves ambiguity particularly in the intermediate-risk group (RS 18–30).The observation that the majority of treatment decision changes resulted in a de-escalation of chemo-endocrine therapy to adjuvant endocrine therapy alone is consistent with other studies. Overall, the proportion of patients for whom chemotherapy was recommended was reduced from 40.3% (89/221) at the first tumor board to 27.1% (60/221) at the second tumor board (a 13.2% net reduction). These data are similar to the findings of the meta analysis from Augustovski et al. [30] and a pooled analysis of four studies by Albanell et al. [31] which reported net reductions in recommendations for chemotherapy of 12% and 13.5%, respectively. In a recent large population-based cohort study of almost 1000 patients from Ontario (Canada) ODX-testing changed the oncologists’ recommendations in half the patients. However this study included a pretest category of “unsure” (whether chemotherapy should be given) which accounted for 328 of 508 patients (65%) who had a change in recommendation. This large study confirms that ODX is far more likely to change the recommendation to omitting chemotherapy (38%) than to recommending it (15%) [32].

Our data are also in agreement with recent recommendations from the 14th St Gallen International Breast Cancer Conference [33]. These guidelines support omitting adjuvant chemotherapy in patients with a low probability of recurrence as determined by multigene expression profiling techniques. At the same time these guidelines caution that adjuvant chemotherapy may be justified in patients with poor pathologic features even if they have a favorable multigene assay result.

Other multi-gene tests and the standardized immunohistochemistry-based scoring system IHC 4 have also been useful in particular to divide patients at intermediate risk for relapse into groups at higher risk and at lower risk [34]. One study suggests that the amount of prognostic information contained in four widely performed immunohistochemical (IHC) assays is similar to that in a multi-gene test [35]. Moreover a study comparing several multi-gene tests to IHC4 (using semiquantitative assessment) showed that the IHC4 score provides better prognostic information than the corresponding quantitative RNA measurements [36]. Thus the combination of quality controlled conventional IHC tests and multigene signatures may provide more information for some patients at intermediate risk of relapse.

Our study has strengths and limitations. Strengths include the prospective nature of the study, prospectively defined endpoints, hypotheses and sample size planning, the formal requirement of the presence of the pathologist at the multidisciplinary tumor-board, the documentation of the multi-step decision-making and implementation process and the trial sponsoring by an academic group (SAKK). On the other hand, observational studies have well-known limitations. When planned prospectively, studies are subject to selection bias. In observational studies investigators will influence study results when they are subject of the investigation and if they are not blinded for the study endpoints and study goals. Furthermore, unobservable and unobserved factors not included in the analyses, are likely to play a role in the patterns and results observed.

## Conclusion

In conclusion, we suggest that patients given a recommendation of HT by a Swiss tumor board may forgo ODX testing, especially if categorized as low risk by the criteria listed in the legend of Table 2. By contrast, in patients recommended CT + HT, knowledge of RS had considerable potential to change treatment recommendations in both risk groups of our patients.

## Appendix

ᅟ

**Table 7:** Characteristics of patients with recommendation change from HT to CT + HT

Variable	Value (*N* = 8)
Recurrence score, median (range)	30 (18–51)
Risk group, *n* (%)	
Low risk	5 (63%)
Non-low risk	3 (38%)
Age, median (range)	61 (44–73)
Menopausal status, n (%)	
Premenopausal	1 (13%)
Postmenopausal	7 (88%)
pT stage, *n* (%)	
T1b	1 (13%)
T1c	6 (75%)
T2	1 (13%)
pN stage, *n* (%)	
pN0	5 (63%)
pN1a	3 (38%)
Histologic type, *n* (%)	
Invasive ductal carcinoma	8 (100%)
Tumor grade (BRE), *n* (%)	
G 2	6 (75%)
G 3	2 (25%)
Peritumoral lympho-vascular invasion, *n* (%)	
No	5 (53%)
Yes	3 (38%)

ᅟ

## Abbreviations

CT + HT: Chemotherapy plus hormonal therapy
HT: Hormonal therapy
LR: Low-risk
NLR: Non-low-risk
ODX: Onco*type* DX®
RC: Recommendation change
RS: Recurrence Score®
SAKK: Swiss Group for Clinical Cancer Research
SERI: Swiss State Secretariat for Education, Research and Innovation
TB: Tumor board
